# A Bamboo Joint-Like Appearance is a Characteristic Finding in the Upper Gastrointestinal Tract of Crohn's Disease Patients: A Case-Control Study

**DOI:** 10.1097/MD.0000000000001500

**Published:** 2015-09-18

**Authors:** Mikihiro Fujiya, Aki Sakatani, Tatsuya Dokoshi, Kazuyuki Tanaka, Katsuyoshi Ando, Nobuhiro Ueno, Takuma Gotoh, Shin Kashima, Motoya Tominaga, Yuhei Inaba, Takahiro Ito, Kentaro Moriichi, Hiroki Tanabe, Katsuya Ikuta, Takaaki Ohtake, Kinnichi Yokota, Jiro Watari, Yusuke Saitoh, Yutaka Kohgo

**Affiliations:** From the Division of Gastroenterology and Hematology/Oncology, Department of Medicine, Asahikawa Medical University (MF, AS, TD, KT, KA, NU, TG, SK, MT, YI, TI, KM, HT, KI, TO, YK); Department of Gastroenterology, Yoshida Hospital (KY); Division of Upper Gastroenterology, Department of Internal Medicine, Hyogo College of Medicine (JW); and Digestive Disease Center, Asahikawa City Hospital (YS).

## Abstract

The clinical importance of Crohn's disease (CD)-specific lesions in the upper gastrointestinal tract (upper GIT) has not been sufficiently established. The aim of this case-control study is to investigate the characteristic findings of CD in the upper GIT.

In 2740 patients who underwent gastroduodenoscopy at Asahikawa Medical University between April 2011 and December 2012, 81 CD patients, 81 gender- and age-matched non-IBD patients, and 66 ulcerative colitis (UC) patients were investigated in the present study. (1) The diagnostic ability and odds ratio of each endoscopic finding (a bamboo joint-like appearance in the cardia, erosions, and/or ulcers in the antrum, notched signs, and erosions and/or ulcers in the duodenum) were compared between the CD and non-IBD patients or UC patients. (2) The interobserver agreement of the diagnosis based on the endoscopic findings was evaluated by 3 experienced and 3 less-experienced endoscopists.

The incidence of detecting a bamboo joint-like appearance, notched signs, and erosions and/or ulcers in the duodenum was significantly higher in the CD patients than in the non-IBD and UC patients. In addition, the diagnostic ability and odds ratio of a bamboo joint-like appearance for CD were higher than those for the other findings. Kendall's coefficients of concordance in the group of experienced and less-experienced endoscopists were relatively high for a bamboo joint-like appearance (0.748 and 0.692, respectively).

A cardiac bamboo joint-like appearance is a useful finding for identifying high-risk groups of CD patients using only gastroduodenoscopy.

## Abstract 2

Supplemental Digital Content is available in the text

## INTRODUCTION

Crohn's disease (CD) is a disease of unclear etiology characterized by chronic inflammation of the entire digestive tract and patients with CD experience chronic abdominal discomfort and pain, diarrhea, and vomiting and abdominal distention when progressing intestinal stenosis. CD is usually diagnosed based on the clinical course, endoscopic results, radiological examinations, and blood tests. Longitudinal ulcers and a cobblestone appearance are typical lesions of CD patients observed during diagnostic imaging.^1–4^ These findings are classified as characteristic findings in the guidelines published by the European Crohn's and Colitis Organization (ECCO) and the Research Group of Intractable Inflammatory Bowel Disease granted by the Ministry of Health, Labour, and Welfare of Japan.^4,5^ However, the incidence of a cobblestone appearance is not so high in CD patients.^6^ Although longitudinal ulcers are common in CD patients, they are frequently observed in patients with other diseases, including ischemic colitis, Behcet's disease, and collagenous colitis. Examinations of small intestine are necessary for the diagnosis of CD in many cases. However, it is difficult to examine the small intestine in all cases with chronic abdominal symptoms because the diagnostic skill of the examinations of the small intestine are not easy and widespread even when using double balloon endoscopy or capsule endoscopy,^7^ thereby causing the delay of the diagnosis of CD. If highly specific and sensitive findings in the upper GIT are determined, in which it is easy to approach with gastroduodenoscopy, it would be helpful for identifying a high-risk group of CD patients from cases with chronic abdominal symptoms, who should undergo the examination of the small intestine.

Lesions of the upper gastrointestinal tract (GIT), including the stomach and duodenum, were first reported by Gottlieb in 1937.^8^ The incidence of these abnormal findings of upper GIT in CD patients had been thought to be much lower than those in the lower GIT (2–3%).^9–11^ However, with the technological and diagnostic progress made in endoscopy, the abnormal findings in the upper GIT of CD patients have been more frequently detected beginning in the 1980s, and now range from 30 to 75%.^12–19^ Typical endoscopic findings in the upper GIT were originally reported to include ulcers and erosions in the antrum and notched signs in the duodenum. In addition, we proposed another endoscopic finding, a bamboo joint-like appearance, as a typical lesion of the stomach in CD patients.^20^ These lesions are characterized by swollen longitudinal folds traversed by erosive fissures or linear furrows and were found in 54% of CD patients.

Subsequently, Hirokawa et al retrospectively investigated the incidence and the histological findings of a bamboo joint-like appearance in the upper GIT of CD patients.^21^ They showed that 15 of 23 (65.2%) CD patients had a bamboo joint-like appearance in their stomach, and the histological features were sharp, deep, and fissure-like erosions associated with lymphoid aggregates, eosinophilic infiltration, and edema at the superficial portion of the surrounding lamina propria. Kuriyama et al investigated the incidence of a bamboo joint-like appearance in the upper GIT in CD patients in comparison to that in the patients with ulcerative colitis (UC) or reflux esophagitis and showed that the lesion was more frequently detected in CD patients than in the patients with either UC or reflux esophagitis.^22^ These studies suggested that the endoscopic findings in the upper GIT, particularly a bamboo joint-like appearance, are characteristic findings in CD patients. However, these previous studies did not address details of the random sequence generation or blinding of the information of the patients before the assessment and did not match regarding the age and the gender between the cases and controls. A reproducibility of the endoscopic findings in upper GIT of CD patients has also been unclear. The present study investigated the incidence of characteristic findings of endoscopy in upper GIT of CD patients, the diagnostic abilities of the endoscopic findings for CD in comparison to non-inflammatory bowel disease (IBD) and UC patients excluded and the inter-observer agreement of the findings among experienced and less-experienced endoscopists.

## PATIENTS AND METHODS

### Patients

**FIGURE 1:**
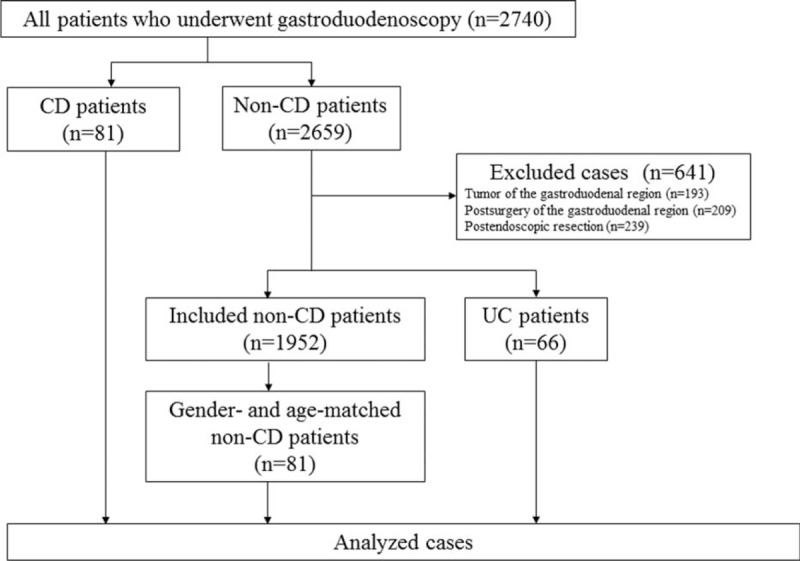
A flow chart of the present study.

From April 2011 to December 2012, 2740 consecutive patients with or without abdominal symptoms underwent gastroduodenoscopy at Asahikawa Medical University. These 2740 examinations did not include any therapeutic procedures, such as either endoscopic mucosal resection or endoscopic submucosal dissection. All patients, including CD patients, underwent gastroduodenoscopy at the time of diagnosis, upon worsening of their symptoms and during the annual follow-up examinations. The endoscopic images of 81 CD patients were used as cases in this case-control study. In the 2659 cases without CD, the patients who had gastroduodenal tumors and a history of either gastroduodenal surgery or endoscopic resection were excluded, because the endoscopic findings could not be sufficiently assessed due to the bleeding from the tumor or deformity caused by multiple scars from the operation(s). Patients with ulcerative colitis were selected as second controls because this disease is considered to be another chronic type of intestinal inflammation that is commonly included in IBD, and some characteristic findings have already been reported to be detected in the upper GIT of UC patients.^22^ Among the remaining 1952 patients, 81 gender- and age-matched patients were randomly selected in order to minimize any bias associated with gender or age (Figure 1). In brief, patient number cards of non-IBD patients were made and divided based on gender and age categories (10–19, 20–29, 30–39, 40–49, 50–59, 60–69, 70–79 and 80–89 years old). The cards of each category were then shuffled in an envelope and thereafter suitable numbers of the cards were blindly selected. All patients were included, regardless of the presence or absence of symptoms. The diagnosis of CD was made based on the combination of the clinical course, colonoscopy, double balloon endoscopy, small bowel enterolysis, and histological findings. All patients who were enrolled underwent gastroduodenoscopy by 1 of 8 physicians (MF, TD, KT, KA, NU, SK, MT, or TI) with endoscopic instruments (GIF-XQ240, GIF-XQ260, GIF-H260Z, Olympus, Tokyo, Japan; EG-530N, Fujinon, Tokyo, Japan) at Asahikawa Medical University Hospital. The physicians were informed that the patients were either CD or non-IBD before performing gastroduodenoscopy. Regardless of the patient's diagnosis, treatment was stopped on the night before the endoscopic examination. A dye-spraying technique with 0.1% indigo carmine was incorporated into the gastroduodenoscopic examination to detect subtle changes. Five to eight pictures demonstrating each part of the stomach, including the cardia, body and antrum, and duodenum were obtained by gastroduodenoscopy regardless of the patient's diagnosis. Biopsies were taken from the patients when they agreed to undergo a histological evaluation. Representative images of the cardia and antrum of the stomach and bulbus of the duodenum in the 81 CD patients and 81 gender- and age-matched non-IBD patients were selected by AS, who did not participate in this image assessment study. No adverse events including either bleeding, which required blood transfusion, or perforation which required a surgical operation to be successfully treated, occurred during the study. Written informed consent was obtained from every participant, and the study was approved by the institutional review board of Asahikawa Medical University (No. 1710).

### Analysis of Characteristic Findings of Endoscopy

**FIGURE 2:**
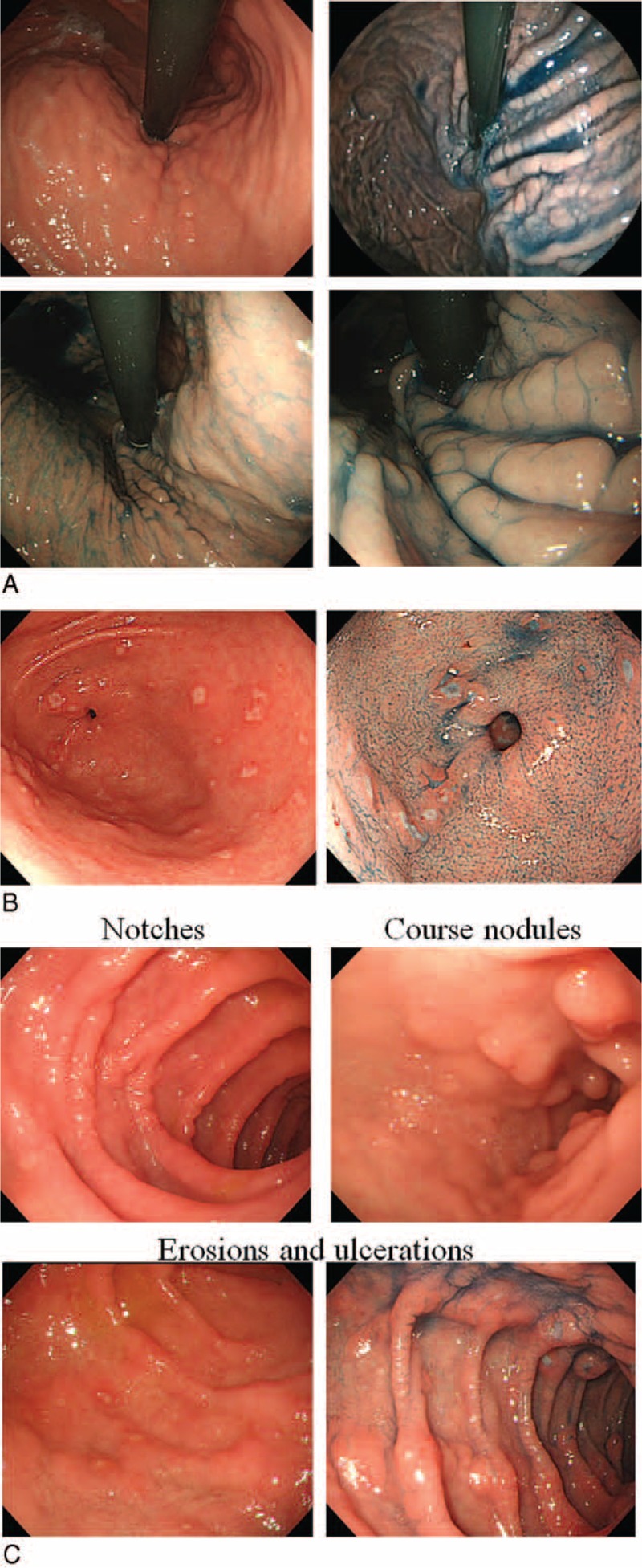
The gastroduodenoscopic findings. Representative images of the bamboo joint-like appearance in the cardia of the stomach (A), erosions/ulcers in the antrum (B) and notched signs, course nodules, and erosions and ulcers in the duodenum (C) are shown.

The endoscopic images of the 81 CD patients and 81 gender- and age-matched non-IBD patients were assessed by a single experienced endoscopist (KM) who had performed >5000 procedures and did not participate in obtaining the images. The images were shuffled and shown to the endoscopist randomly with no information regarding the patient, including demographics, medical examination findings, or the diagnosis. The incidence of the detection of endoscopic features in the upper GIT, including a bamboo joint-like appearance in the cardia (Figure 2A), erosions and/or ulcers in the antrum (Figure 2B), notched signs, and erosions and/or ulcers in the duodenum (Figure 2C), was examined, and the diagnostic accuracy, sensitivity, specificity, and odds ratio of each endoscopic feature were calculated. The incidence of the detection of endoscopic features was compared with Crohn's Disease Activity Index (CDAI), the extension of disease, treatments, and the *Helicobacter pylori* (HP) infection status that was determined by the anti-HP IgG antibody titer, histological examinations, and culture tests in 49 patients who agreed to undergo the tests. Among the 49 CD patients, only the serum anti-IgG antibody was examined in 31 patients, both the serum anti-IgG antibody and bacterial culture in 7 patients, both the serum anti-IgG antibody and histological assessment of biopsy in 1 patients, both the bacterial culture and histological assessment in 9 patients, and only histological assessments were performed in 1 patient.

### Inter-Observer Agreement

The images of the 81 CD patients were shown to 6 physicians, including 3 experienced (MF, YI, NU) and 3 less-experienced endoscopists (KT, MI, TO). The experienced and less-experienced endoscopists were defined as physicians who had performed >5000 and <500 endoscopic examinations, respectively.^23^ These physicians diagnosed the presence or absence of endoscopic findings including a bamboo joint-like appearance of the cardia, notched signs, and erosion or ulceration of the duodenum. The inter-observer agreements in the groups of experienced and less-experienced endoscopists were separately examined.

### Statistical Analysis

Fisher's exact test was applied for the statistical analysis of the characteristic findings of endoscopy and the correlations between the incidence of the endoscopic findings and HP infection, extension of disease, and treatment. Kendall's coefficient of concordance was used for the statistical analysis of the inter-observer agreement. Student's *t* test was used to compare the endoscopic findings and CDAI. A *P* value < 0.05 was considered to be statistically significant.

## RESULTS

### The Incidences and Diagnostic Abilities of the Endoscopic Findings

To determine whether the endoscopic findings were characteristic for the patients with CD, the detection rates of the endoscopic findings were compared between the CD patients and non-IBD patients. The demographic data for the CD patients are shown in Table 1. There were no missing data and no outliers for the index test in this study. The detection rates of a bamboo joint-like appearance in the cardia and erosions and/or ulcers in the antrum were 38.3% and 46.9% in the CD patients and 2.5% and 33.3% in the gender- and age-matched non-IBD patients, respectively. In the duodenum, notched signs and erosions and/or ulcers were detected in 9.9% and 32.1% in the CD patients, 6.2% and 22.2% in the gender- and age-matched non-IBD patients, respectively. The incidence of a bamboo joint-like appearance in the stomach was significantly higher in the CD patients than in the gender- and age-matched non-IBD patients (*P* < 0.0001). The detection rate of a bamboo joint-like appearance in the cardia, erosions and/or ulcers in the antrum, notched signs, and erosions and/or ulcers in the duodenum of UC patients were 1.5%, 19.7%, 1.5%, and 4.5%, respectively. The detection rates of these all findings were significantly higher in CD patients than those in UC patients (Table 2). Of the 81 CD patients, 64 patients (79.0%) had 1 or more lesions, including a bamboo joint-like appearance, erosion/ulcer in the stomach, notches and erosions/ulcers in duodenum. Seventeen patients (21.0%) had no lesions. Although biopsies were taken from 15 of the 31 patients with lesions with a bamboo joint-like appearance, 6 of the patients with 38 erosions/ulcers in the stomach, 1 of 8 patients with notches and 1 of 26 patients with erosions/ulcers in duodenum, no granuloma was histologically detected in any of our patients examined.

**TABLE 1:**
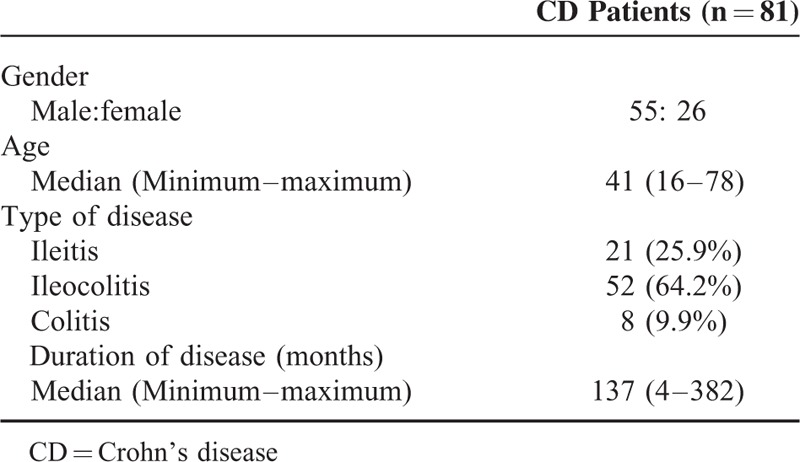
The Demographics of the CD Patients

**TABLE 2:**
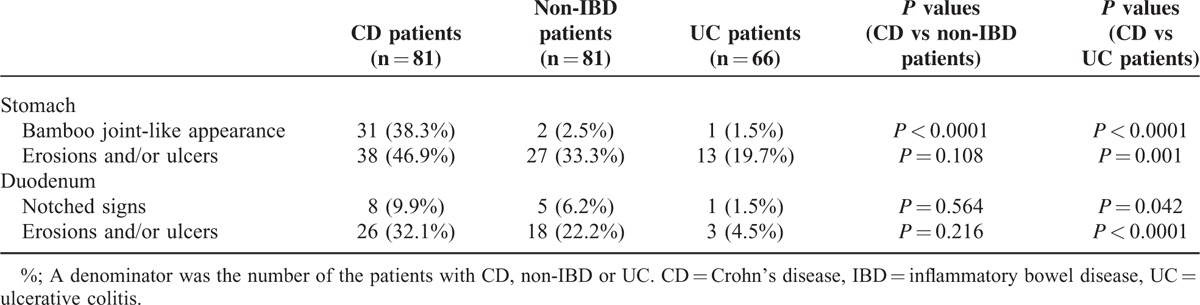
The Incidence of the Endoscopic Findings in the CD Patients, Gender- and Age-Matched Non-IBD Patients, and UC Patients

Based on these results, the diagnostic abilities of these four endoscopic findings were calculated. The diagnostic accuracies, sensitivities, and specificities for diagnosing CD in comparison to the gender- and age-matched non-IBD patients were 67.9%, 38.3%, and 97.5% for a bamboo joint-like appearance in the stomach, 56.8%, 46.9%, and 66.7% for erosions and/or ulcers in the stomach, 51.9%, 9.9%, and 93.8% for notched signs in the duodenum and 54.9%, 32.1%, and 77.8% for erosions and/or ulcers in the duodenum, respectively. The odds ratios of these 4 findings for CD were 24.49 (95% confidence interval; 5.61–106.85), 1.77 (0.94–3.34), 1.67 (0.52–5.33), and 1.65 (0.82–3.34), respectively (Table 3). The diagnostic accuracy and specificity of a bamboo joint-like appearance were higher than those of other endoscopic findings, and the odds ratio of only a bamboo joint-like appearance for CD was significantly high, indicating that the detection of a bamboo joint-like appearance is a useful marker for diagnosing CD using gastroduodenoscopy. No correlations between the detection rate of each feature and the CDAI (Supplemental Table 1, http://links.lww.com/MD/A408), extension of disease (Supplemental Table 2, http://links.lww.com/MD/A408), or HP infection status were observed (Supplemental Table 3, http://links.lww.com/MD/A408). The detection rate of a bamboo joint-like appearance was significantly lower in CD patients receiving 5-ASA treatment (31.7%) in comparison to that in the patients with no 5-ASA treatment (61.1%). The detection rate of erosions and/or ulcers in the duodenum was significantly higher in CD patients who used infliximab (IFX) or adalimumab (ADA) (44.1%) than that in patients without the administration of IFX or ADA (18.4%) (Supplemental Table 4, http://links.lww.com/MD/A408).

**TABLE 3:**
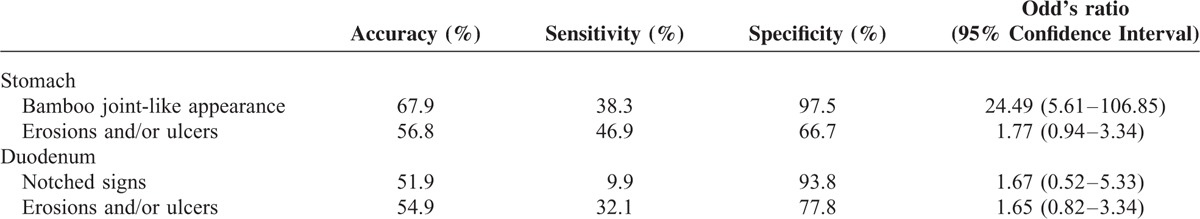
The Diagnostic Accuracy and Odds Ratio of each Endoscopic Finding (vs non-IBD Patients)

### Inter-Observer Agreement Study

To elucidate the inter-observer agreements of the endoscopic features, the images of the cardia in the stomach and bulbus in the duodenum were separately assessed by 3 experienced and 3 less-experienced endoscopists. Kendall's coefficient of concordance in the experienced, less-experienced, and all endoscopists were 0.748, 0.692, and 0.637 for a bamboo joint-like appearance, 0.450, 0.514, and 0.384 for notched signs in the duodenum, and 0.657, 0.671, and 0.565 for erosions and/or ulcers in the duodenum, respectively. Kendall's coefficient of concordance for a bamboo joint-like appearance was >0.6 in all groups, illustrating that there was a good reproducibility of the findings among endoscopists with various levels of experience (Table 4).

**TABLE 4:**
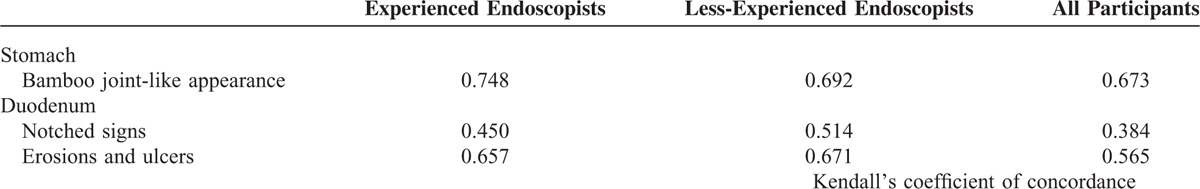
The Inter-Observer Agreement for Each Endoscopic Finding

## DISCUSSION

The present study is the first study to assess the diagnostic value of various features for detecting upper GIT lesions in CD patients and demonstrated that a bamboo joint-like appearance is a characteristic finding in the upper GIT in CD patients, with a diagnostic accuracy of 68% among gender- and age-matched non-IBD patients, as determined from a case-control study. The odds ratio of the presence of a bamboo joint-like appearance was extremely high (24.49 [95% confidence interval; 5.61–106.85]). The detection rate of a bamboo joint-like appearance in CD patients was significantly higher in patients with UC, which is another type of IBD. Furthermore, it is noteworthy that the inter-observer agreements of the bamboo joint-like appearance were sufficiently high (> 0.6) among both experienced and less-experienced endoscopists, suggesting that a bamboo joint-like appearance is a useful marker for identifying a high-risk group of CD patients by gastroduodenoscopy, who should undergo the examination of the small intestine, and that it can be used regardless of the experience levels of the endoscopist.

Longitudinal ulcers and a cobblestone appearance are known as typical lesions of CD.^1–5^ Hisabe et al reported that 81.7% and 41.1% of CD patients have longitudinal ulcers and a cobblestone appearance, respectively.^6^ Although longitudinal ulcers are common in CD patients, they are sometimes seen in patients with ischemic colitis, Behcet's disease, and collagenous colitis. The sensitivity of the bamboo joint-like appearance is lower than that of longitudinal ulcers, but almost equivalent to that of a cobblestone appearance. A bamboo joint-like appearance is therefore a useful marker for diagnosing CD, in addition to the presence of longitudinal ulcers and a cobblestone appearance.

As we first reported the high incidence of a bamboo-joint-like appearance detected in the stomach of CD patients,^20^ 2 studies and some case reports suggested the usefulness of a bamboo joint-like appearance for diagnosing CD.^21,22,24–26^ Hirokawa et al showed in their retrospective study that a bamboo joint-like appearance in the upper GIT was detected in 15 of 23 (65.2%) CD patients.^21^ Kuriyama et al also showed that there was a high incidence of the bamboo joint-like appearance (28 of 63 [44%]) in CD patients in comparison to that in the patients with UC (5%) or reflux esophagitis (0%).^22^ However, it was not clearly shown in these previous studies whether the clinical status of the patients was blinded to the assessors, and the control cases in these studies included patients with specific diseases, such as UC and reflux esophagitis. The present study demonstrated a higher incidence (38.3%) of a bamboo joint-like appearance in CD patients than in gender- and age-matched non-IBD patients (2.5%) who were likely to be members of the general population undergoing gastroduodenoscopy. Taken together, these data indicate that a bamboo joint-like appearance is frequently detected in CD patients, but is rarely observed in patients with other diseases and healthy population.

The etiology of the bamboo joint-like appearance is still unclear. Hirokawa et al analyzed the histological findings of the biopsy specimens obtained from lesions with a bamboo joint-like appearance and proposed that microfissures, including sharp fissure-like erosions and/or mucosal clefts, were demonstrated in 7 of 14 (50%) patients and a granuloma was detected in 2 of the 14 (14.3%) patients in the histological specimens obtained from the lesion with a bamboo joint-like appearance in the stomach of CD patients.^21^ In this study, no granuloma was detected by an upper GIT series. It is known that, even in the colon, the detection rate of granuloma in typical lesions tends to be low.^27–29^ These low detection rates of granuloma might have been due to the fact that the histological findings were diagnosed using only mucosal samples obtained by endoscopic biopsy. The present study also showed that HP infection was not associated with the presence of a bamboo joint-like appearance. Matsumura et al has also reported that a bamboo joint-like appearance was frequently detected in the HP-positive group as well as in the HP-negative group.^30^ This suggests that the bamboo joint-like appearance is not a type of chronic gastritis due to HP infection. While the high incidence of the detection rate of the bamboo joint-like appearance, the etiology of the lesion has been unclear. Further large-scale studies with a molecular biological analysis are warranted to clarify the pathogenesis of the bamboo join-like appearance.

Concerning the disease activity, the present study revealed no correlation between the CDAI and the incidence of detecting a bamboo-joint-like appearance. Hirokawa et al showed a higher detection rate of a bamboo-joint-like appearance in CD patients with a high level of CRP (>0.3 mg/dl) than in those with a lower CRP level, but not the association between the presence of a bamboo joint-like appearance and the activities of the lesions in other sites.^21^ Consequently, a bamboo joint-like appearance is useful for predicting the presence or absence of CD regardless of the severity of CD. In this study, the detection rates of a bamboo joint-like appearance and erosions and/or ulcers in the duodenum were influenced by the presence or absence of the administration of 5-ASA and anti-TNFα treatment, respectively. However, the treatments were selected by physicians based on the condition and disease activity of the patients, with no randomization, thus suggesting that the selection of the treatments may have included a large bias. These results were therefore not thought to be reliable.

Although there were no significant differences in the incidence of erosions and/or ulcers in the stomach and duodenum or notched signs, the present study demonstrated that the incidence of these features in CD patients is higher than that observed in gender- and age-matched non-IBD patients, and that HP infection was not associated with the incidence of these gastric erosions and/or ulcers and duodenal lesions in CD patients. The result is consistent with previous investigations.^19,30–33^ However, the odds ratios of detecting these 3 findings were much lower than those associated with the bamboo joint-like appearance, suggesting that such lesions are common in non-IBD patients. In order to use these lesions as markers for the diagnosis of CD, morphological characteristics which are specific for CD-related lesions should be determined.

This study is associated with several potential limitations. First, the study was a retrospective case-control single-center study. Second, the endoscopists who performed the gastroduodenoscopies were informed about the diagnosis of the patients at the examination, while the endoscopists who assessed the endoscopic images were not informed about the patients, including their demographics, medical examination findings, or the diagnosis. Third, only 1 participant selected the images to assess endoscopic findings. However, at our institute, we routinely take pictures of each part of the stomach and duodenum at gastroduodenoscopy regardless of the patient's diagnosis. This might have reduced the bias when picking up and selecting pictures. Fourth, *HP* infection was assessed using different procedures in each case. Further blinded prospective studies are needed to evaluate the diagnostic value of the characteristic findings.

In summary, the present study demonstrated that a bamboo joint-like appearance in the cardia of the stomach is a characteristic finding on gastroduodenoscopy in CD patients. The finding might be useful for identifying a high-risk group of CD patients using only gastroduodenoscopy, before the examination of the small intestine. Because of the high inter-observer agreement of the presence of a bamboo joint-like appearance among endoscopists with various levels of experience, the finding was thought to be an applicable marker for CD by experienced, as well as less-experienced, endoscopists.
